# Vipers of the Middle East: A Rich Source of Bioactive Molecules

**DOI:** 10.3390/molecules23102721

**Published:** 2018-10-22

**Authors:** Mohamad Rima, Seyedeh Maryam Alavi Naini, Marc Karam, Riyad Sadek, Jean-Marc Sabatier, Ziad Fajloun

**Affiliations:** 1 Department of Neuroscience, Institut de Biologie Paris Seine (IBPS), INSERM, CNRS, Sorbonne Université, F-75005 Paris, France; maryam.alavi.naini@gmail.com; 2 Department of Biology, Faculty of Sciences, University of Balamand, Kourah3843, Lebanon; marckaram1@gmail.com; 3 Department of Biology, American University of Beirut, Beirut 1107-2020, Lebanon; rsadek@aub.edu.lb; 4 Laboratory INSERM UMR 1097, Aix-Marseille University, 163, Parc Scientifique et Technologique de Luminy, Avenue de Luminy, Bâtiment TPR2, Case 939, 13288 Marseille, France; sabatier.jm1@libertysurf.fr; 5 Department of Biology, Faculty of Sciences III, Lebanese University, Tripoli 1300, Lebanon; 6 Laboratory of Applied Biotechnology, Azm Center for Research in Biotechnology and Its Applications, EDST, Lebanese University, Tripoli 1300, Lebanon

**Keywords:** *Montivipera bornmuelleri*, *Macrovipera lebetina*, *Vipera (Daboia) palaestinae*, snake venom, viper

## Abstract

Snake venom serves as a tool of defense against threat and helps in prey digestion. It consists of a mixture of enzymes, such as phospholipase A2, metalloproteases, and l-amino acid oxidase, and toxins, including neurotoxins and cytotoxins. Beside their toxicity, venom components possess many pharmacological effects and have been used to design drugs and as biomarkers of diseases. Viperidae is one family of venomous snakes that is found nearly worldwide. However, three main vipers exist in the Middle Eastern region: *Montivipera bornmuelleri*, *Macrovipera lebetina*, and *Vipera (Daboia) palaestinae*. The venoms of these vipers have been the subject of many studies and are considered as a promising source of bioactive molecules. In this review, we present an overview of these three vipers, with a special focus on their venom composition as well as their biological activities, and we discuss further frameworks for the exploration of each venom.

## 1. Snake Venom: An Overview

### 1.1. Types and Functions

Venomous species mainly belong to the kingdom, “Animalia”; the most studied terrestrial ones are snakes, scorpions, and spiders. Snakes are reptiles belonging to the suborder, “Serpentes”. They are found on every continent except Antarctica. Venom study traces back to Aristotle (384–322 BC Aristotle “Historia Animalium”), in which he detailed venomous animals and their bites. Francesco Redi, one of the founders of toxicology, found that toxicity is in the venom itself rather than in the snake bile, as was previously accepted. Snake venom glands were discovered during the 18th century and were experimentally used by Felice Fontana [1]. The importance of venom studies stems from the fact that 1.8–2.5 million venomous snake bites are reported worldwide every year, according to many trust worthy reports, with over 100,000 bites resulting in fatalities [2]. Other than serving as a tool for protection against an attack, venom components also aid in the immobilization and the digestion of the prey. In fact, it has been shown that combining bovine muscle with particular venoms could enhance the digestion of the muscle tissue [3].

Venoms are classified according to their toxic effects: Hemotoxic venoms act on the cardiovascular system and blood functions by targeting blood cells and disrupting blood clotting, which leads to severe pain, internal bleeding, and damage to various organs. Cardiotoxins, for instance, can interact with membrane proteins of cardiomyocytes, cause their depolarization, and thus prevent contraction [4]. Venom compounds harming the nervous system are called neurotoxins and act by inhibiting ion channels, therefore, impairing ion flow across membranes or by mimicking acetylcholine, and thus impairing communication between neurons [5]. Since the nervous system is involved in the regulation of body functions, this type of venom is considered to be the deadliest [5]. Cytotoxic venom targets body cells, causing necrosis; some cytotoxins, such as phospholipases, interact with the cell membrane, leading to the disruption of the phospholipid bilayer and the formation of pores [6]. Each snake has a different combination of these damaging toxins, and therefore has developed several protective mechanisms against their own venom. For example, *N*-glycosylation of nicotinic Acetylcholine receptors’ (nAChR) binding site confers resistance against conspecific α-neurotoxin [7]. Circulating antibodies neutralizing venom components is another form of self-protection that snakes have evolved [8]. A study revealed that acidification of stored venom components by the mitochondria-rich cells, found in the main gland of rattlesnakes, inhibits the venom’s enzyme activities and thus allows the long-term storage and the on-demand use of the venom [9].

### 1.2. Snake Venom Components

Snake venom contains a mixture of mostly enzymes and non-enzymatic proteins or peptides constituting 90 to 95% of the venom’s dry weight. Other components include carbohydrates, lipids, metal ions, and inorganic anions. The most common enzymes include phospholipase A2 (PLA2), hyaluronidase, metalloproteases, l-amino acid oxidase (l-AAO), acetylcholinesterases, and serine proteases [10].

Snake phospholipases A2 belong to the superfamily of secreted PLA2s. These multi-toxic enzymes catalyze the hydrolysis of membrane phospholipids in erythrocytes and various cells causing membrane lesions and direct hemolysis [11]. PLA2s that affect skeletal muscles are called myotoxic and act by disrupting the membrane potential and structure, and thus can promote muscle necrosis [12]. Furthermore, snake PLA2s are neurotoxic [13], bactericidal [14], and have shown pro-inflammatory activity [15]. In addition, they are able to disturb the hemostatic cascade by exhibiting anticoagulant effects and by affecting platelets’ function.

Some snake venoms are also rich in metalloproteases, which are zinc-dependent enzymes that degrade proteins of the extracellular matrix and components of the hemostatic system and thus cause local and systemic bleeding by disrupting microvessels [16]. In fact, snake hemorrhagins have been found to be metalloproteases, targeting mainly the basement membrane proteins underlying the capillaries endothelial cells [17]. Snake venom metalloproteases are also involved in the pathogenesis of tissue damage and edema. Other functions include the disruption of platelet adhesion and aggregation, fibrinolytic activity, prothrombin activation, apoptosis, and inflammation [18,19,20,21,22]. Anai et al. showed that venom’s antigens were higher in the plasma of rats given crude venom as compared to rats given hemorrhagic metalloprotease-neutralized venom [23]. This implicates a role for metalloproteases in the spreading of venom components, especially coagulation factors, from the site of bite into systemic circulation.

Acetylcholinesterases catalyze the hydrolysis of acetylcholine to choline and acetic acid. They are found particularly in the family of Elapidae [24]. Acetylcholinesterases affect the nervous system by hydrolyzing acetylcholine and thus relax the muscles and cause rapid termination of nerve impulse transmission in the cholinergic system [5].

Hyaluronidases are glycosidases capable of degrading hyaluronic acid, a polysaccharide found in the extracellular matrix (ECM) of mainly soft connective tissue [25]. This leads to the destruction of ECM integrity in these tissues surrounding blood vessels and smooth muscles that help in the diffusion and distribution of toxins. Therefore, both hyaluronidases and metalloproteases are considered to be venom spreading factors. Hyaluronidase inhibitors are not only able to reduce local tissue damage, but also systemic toxicity by preventing venom toxins in reaching circulation [26].

l-amino acid oxidases (l-AAO) catalyze the oxidative deamination of l-α amino acid to give α-keto acid with production of ammonia and hydrogen peroxide. l-AAOs induce local alterations, such as hemorrhage and edema. In 1982, Nathan et al. found that l-AAO from *Echis colorata* venom impairs platelet aggregation [27], whereas Li et al. suggested that l-AAO purified from king cobra *Ophiophagus hannah* venom causes human platelet aggregation [28]. Other systemic alterations include an anti-coagulant effect. Furthermore, snake venom l-AAOs display antibacterial [29], antiviral [30], anti-parasitic, and apoptosis inducing activities [31].

Serine proteases cleave covalent peptide bonds in proteins. They are able to disturb hemostasis and thrombosis through their possession of fibrinolytic activities. Thrombin-like enzymes are serine proteases that clot fibrinogen and form loose fibrin clots that can be quickly degraded. Snake venom serine proteases can also activate plasminogen, leading to its conversion into plasmin [32,33], activate blood coagulation factors, activate platelet aggregation, and exhibit kininogenase activity, leading to the release of bradykinin.

Non-enzymatic peptides of venom include desintegrins that inhibit integrin-receptor binding and thus inhibit platelets’ aggregation. They also induce apoptosis of endothelial cells [34]. Bradykinin-potentiating peptides (BPP) activate bradykinin, a peptide causing the dilation of blood vessels [35]. Natriuretic peptides along with BPP are hypotensive agents [36]. Hemextins AB, from the venom of *Hemachatus haemachatus*, are three-finger toxins, which are also non enzymatic proteins of snake venom that exhibit a synergistically enhanced anticoagulant activity by inhibiting blood factor FVIIa and exposed tissue factor (TF) involved in blood clotting [37].

## 2. Snake Venom Uses

### 2.1. Medicinal Applications

Although being dangerous, venomous animals, like snakes, have always been associated with healing. Indeed, the symbol of medicine is the Rod of Asclepius, commonly mistaken for the Caduceus. It illustrates a staff with a sacred snake coiled around it and represents healing and renewal. Beside its role in prey acquisition and defense, venom components form a pool of pharmaceutical products are applicable in medical practice and drug discovery. Several snake venom components have been studied for their antimicrobial effects. Venoms from different snake species, in particular those of Vipiridae and Elapidae, two families belonging to the suborder, Serpentes, exhibit strong antimicrobial effects against Gram positive and Gram negative bacteria, with those of Vipiridae showing a broad spectrum of activity that may result from the presence of PLA2 and l-AAO enzymes [38]. Crotamine, a myotoxin from *Crotalus durissus*, kills *E. coli* by increasing the permeability of the bacterial membrane in vitro [39]. Regarding liver diseases, snake venom preparation from *Agkistrodon halys pallas* was found to be beneficial in a rat model of fibrotic/cirrhotic liver disease where treated animals demonstrated enhanced bile flow and hepatic microcirculation among other improvements [40]. As to its analgesic effect, in 1995, Pu et al. characterized a neurotoxin from the venom of king cobra *Ophiophagus hannah* with analgesic action and named it hannalgasin [41]. Snake venom components have also been described as exhibiting antitumor activity. This hypothesis was first tested in 1993 by Calmette, who found that toxins from *Vipera lebetina turnica* lead to apoptosis of ovarian cancer cells in mice models [42]. In addition, Cathelicidin-BF, an antibacterial peptide from the venom of *Bungarus fasciatus*, suppressed proliferation and angiogenesis of a metastatic melanoma cell line both in vitro and in vivo [43]. One could argue that the venom might destroy healthy cells along with cancerous cells; for that reason, snake venom delivery systems are being developed to directly target and suppress tumor cells using nanotechnologies, such as combining the venom with silica nanoparticles [44].

### 2.2. Drugs Based on Venom Components

The use of snake venom in pharmaceutical drug discovery is gaining interest. As such, different drugs based on venom components have been Food and Drug Administration (FDA) approved, marketed in some countries, or still in clinical trials.

Captopril is the first drug designed based on a snake venom peptide, a bradykinin potentiating peptide from the pit viper *Bothrops jararaca.* The drug is an angiotensin-converting enzyme inhibitor that serves for the treatment of hypertension and some forms of congestive heart failure by blocking the generation of angiotensin II and inhibiting the degradation of bradykinin [35,45]. Tirofiban and Eptifibatide are two anti-platelet drugs introduced simultaneously in 1998. Tirofiban is a synthetic anti-coagulant drug mimicking a disintegrin found in the venom of *Echis carinatus*, and Eptifibatide is a synthetic heptapeptide mimicking the action of a disintegrin found in the *Sistrurus miliarius barbouri* venom [46,47]. They inhibit the GpIIb/IIIa receptor on the surface of platelets and thus prevent platelet aggregation by competing with fibrinogen for receptor binding. Batroxobin, which is the main compound in *Bathrops atrox* venom, renders the blood more prone to coagulation. This conclusion was elaborated after patients undergoing hip replacement surgery while receiving batroxobin showed less perioperative blood loss than in the control group [48]. Hemocoagulase, an enzyme complex that is found in the venom of *Bothrops atrox*, was shown to prevent and treat hemorrhage. In fact, preterm infants with pulmonary hemorrhage receiving Hemocoagulase showed reduced duration of hemorrhage and a lower mortality risk as compared to its use as a prophylactic agent [49]. Hemocoagulase is therefore used for topical wound healing [50].

## 3. The Viperidae Snake Family

Viperidae is a family of extensively studied venomous snakes that exist in a wide range of habitats. Vipers are characterized by long hollow venom-injecting fangs that can be folded back when not in use, a triangular head distinct from the neck, a stocky body with keeled scales, and vertically elliptical pupils [9]. Its subfamilies include Viperinae, Crotalinae, Causinae, and Azempiopinae. 

**Figure 1:**
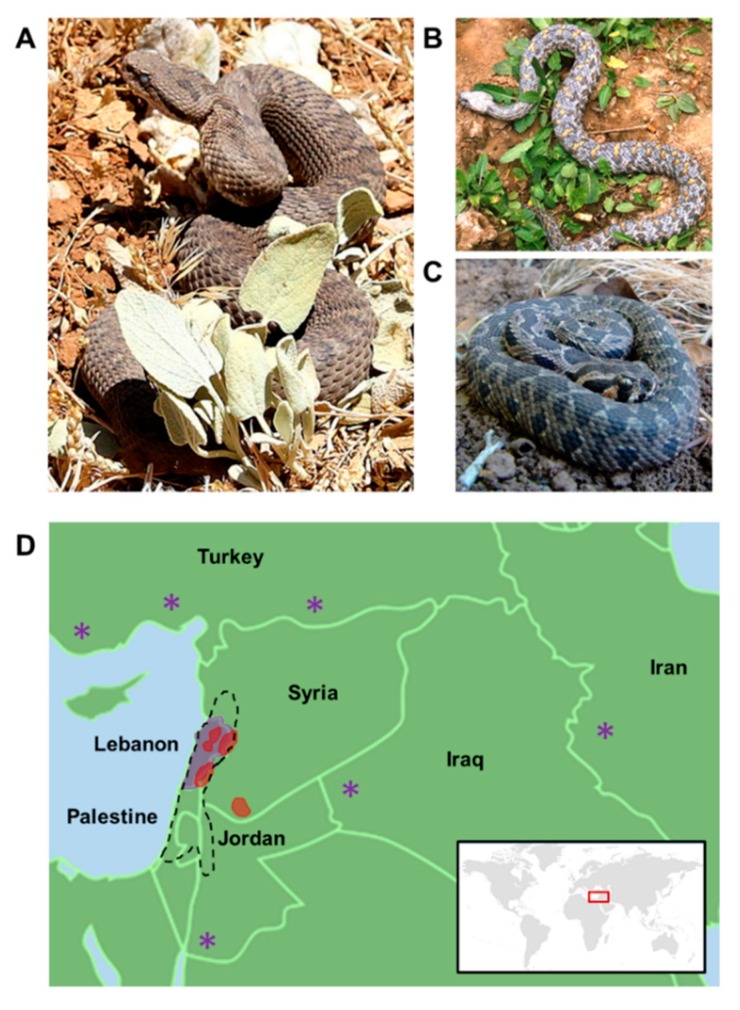
Main vipers of the Middle East region. Photos of (**A**) *Montivipera bornmuelleri* captured by Mickey Samuni-Blank^©^, (**B**) *Macrovipera lebetina* captured by Jan Ševčík^©^, and (**C**) *Vipera (Daboia) palaestinae* captured by Guy Haimovitch^©^. (**D**) Geographic distribution of *Montivipera bornmuelleri* (in red), *Macrovipera lebetina* (in magenta), and *Vipera (Daboia) palaestinae* (in black). *Montivipera bornmuelleri* is present in Lebanon, Syria, and Palestine (red sector). *Macrovipera lebetina* is very common in Lebanon (magenta sector), but rare in Jordan, Iraq, Iran, and Turkey (magenta asterisks). Subspecies of *Macrovipera lebetina* can also be found in Tunisia, Cyprus, and Algeria. *Vipera (Daboia) palaestinae* is mostly found in Lebanon, Syria, Palestine, and Jordan (dashed black sector). Vipers’ distribution was represented based on ‘The IUCN (The International Union for Conservation of Nature) red list of threatened species’ data.

Viperidae venom is a proteolytic venom containing an abundance of proteases that cause pain, strong local swelling, and perturbation of the normal haemostatic system [17]. Each venom has predominant effects depending on the family. Elapidae, another family of venomous snakes, is characterized by a venom that is mainly neurotoxic, whereas Viperidae venom, due to its effect on the vascular system, is classified as being hemotoxic. Consequently, Viperidae envenomation leads to persistent bleeding and collapse in blood pressure [51]. Although vipers’ venoms are hemotoxic, some exceptions exist; in some instances, it can result in neurotoxicity [52]. Three viper species have been recorded in the Middle East: *Montivipera bornmuelleri*, *Macrovipera lebetina*, and *Vipera (Daboia) palaestinae* (Figure 1). The venoms of these vipers have been the subject of many studies and therefore can be considered as a promising source of molecules with therapeutic interest. In this review, we present an overview of these three vipers, with a special focus on their venoms’ compositions as well as their biological activities.

## 4. *Montivipera bornmuelleri*

*Montivipera bornmuelleri* (Figure 1A), also known as the Lebanon viper, is a venomous snake belonging to the family of Viperidae and to the subfamily of Viperinae. This species is endemic to high altitudes (above 1800 m); it is found mainly in Lebanon and Syria, and specifically in the Mount Lebanon range and Mount Hermon, and less abundantly in Palestine (Figure 1D). These snakes are ovoviviparous and breed once a year with births occurring between August and September [53]. Members of the *Montivipera* genus are usually short tailed and grow to a maximum length of 75 cm. The IUCN red list of threatened species classified *M. bornmuelleri* as being endangered due to overgrazing of habitat, the use of off-road vehicles, and the use of its habitat for military purposes or to the development of the skiing industry [54].

### 4.1. Venom Composition

*Montivipera bornmuelleri* venom composition has been evaluated using different analytical techniques. The proteomic analysis showed that *M. bornmuelleri’s* venom contains 65 protein compounds. According to previously established molecular masses of toxins and enzymes in snake venom, it was proposed that *M. bornmuelleri* contains protein families corresponding to serine proteases, phospholipases A2 (PLA2), and metalloproteases III, which comprise at least 30% of the total venom of most vipers [55]. However, N-terminal sequencing of these compounds is still required and essential for a definitive assignment of the different proteins found in this viper’s venom. Among these components, PLA2 and l-AAO have been characterized. PLA2 was purified in 2014 by combining two purification procedures: Size-exclusion chromatography on Biogel P60 and reverse high pressure liquid chromatography (HPLC) on Restek Ultra II C_18_ column. In the same year, we described l-AAO as another compound present in *M. bornmuelleri’s* venom. The enzyme was purified by size-exclusion chromatography and HPLC, and the identity of l-AAO was validated by enzymatic activity testing of the suspected l-AAO-containing fraction [56].

The identified compounds showed interesting biological activities that will be described in the following paragraph, and therefore need more investigation for therapeutic applications. In addition, a wide list of potential enzymes and toxins remains poorly identified in *M. bornmuelleri’s* venom and should therefore be explored.

### 4.2. Venom Biological Activities

Many studies have focused on the biological properties of *M. bornmuelleri’s* crude venom mostly in vitro; however, some studies were conducted in vivo. Accary et al. showed that the venom possesses antibacterial activity against Gram-positive and Gram-negative bacteria, with the most significant effect on *Staphylococcus aureus* and *Morganella morganii*, and an intermediate activity against the fungus, *Candida albicans* [55]. The same team also showed that *M. bornmuelleri* venom disturbs the coagulation cascade by either showing pro- or anti-coagulant activities at different concentrations in human plasma [57]. This is consistent with the fact that the venom of the Viperidae family of snakes is widely studied for its pro-coagulant and anticoagulant properties [58]. A study conducted on the effect of *M. bornmuelleri* on human blood showed that the venom was unable to induce direct lysis of red blood cells (RBCs) in vitro; however, dose-dependent hemolysis was observed in the presence of lecithin [57]. Results suggest that *M. bornmuelleri’s* venom lacks direct lytic factors, but is able to induce indirect hemolysis due to the presence of PLA2 that are able to hydrolyze lecithin. To further characterize the role of PLA2 in this Lebanese viper venom, the purified enzyme was tested for its biological activity. It has been shown that *M. bornmuelleri’s* PLA2 exhibits strong anti-bacterial, hemolytic, anti-coagulant, and pro-inflammatory activities [59]. This is in accordance with previous studies elucidating the effects of the crude venom of *M. bornmuelleri* as well as studies revealing the varied properties of PLA2. l-AAO was another major component purified from *M. bornmuelleri* venom that was also found to exhibit antibacterial activity against Gram-negative bacteria. Its potential therapeutic use stems from the fact that these l-AAOs are not cytotoxic to human erythrocytes [56].

Viper venom is known to reduce blood pressure; this is also true for *M. bornmuelleri’s* venom. In fact, the venom displays vasorelaxant effects by acting synergistically on different pathways. It can act on endothelial cells and induce the release of the vasoactive mediator, NO, reduce Ca^2+^ influx through voltage-dependent calcium channels, and inhibit contraction induced by angiotensin I [60]. In this study, they suggest that the observed effect is due to PLA2 and metalloproteinases present in the venom; however, further work is needed to identify and purify the exact compounds that are responsible for the vasorelaxant effects.

More recently, the anti-cancer potential of *M. bornmuelleri’s* venom has been evaluated by testing its toxicity on tumorigenic and non-tumorigenic cell lines [61]. In this study, it was shown that *M. bornmuelleri’s* crude venom selectively exhibits a cytotoxic effect depending on the cell line type. In fact, *M. bornmuelleri’s* crude venom is more toxic on benign and malignant cells (A5 and II4 cells, respectively) than non-tumorigenic HaCaT cells. The selective cytotoxic effect may be due to an interference of some venom compounds with potential signaling pathways that are up-regulated in cancer cells. These findings highlight the possible potential of *M. bornmuelleri* in cancer treatment and open the horizon for more studies investigating anti-cancer molecules within the venom as well as their mechanism of action. It is important to mention that most of the above-stated results have been obtained in vitro; therefore, in vivo studies should also be considered. These experiments are now feasible since the toxicity of the venom has been recently identified in vivo [62]. More recently, the immunomodulatory effects of *M. bornmuelleri* venom in vivo on the splenic levels of tumor necrosis factor, TNF-α; Interferon IFN-γ; and Interleukins, IL-4, IL-10, IL-1β, and IL-17 have been studied in vivo [63]. In fact, *M. bornmuelleri* venom up-regulated the levels of the pro-inflammatory cytokines, TNF-α, IFN-γ, IL-1β, and IL-17, and established a trend in decreasing the anti-inflammatory cytokine, IL-10. By shifting inflammation towards a dominant activated T helper cells (Th1/Th17) rather than a T helper cell type 2/T regulatory cell (Th2/Treg) response, the venom of interest may activate anti-tumor immunity and break tumor tolerance.

On the other hand, it is well known that vipers’ venoms could act on either the vascular system or the nervous system. Nevertheless, no study until today investigated the effect of *M. bornmuelleri’s* venom on the nervous system. Therefore, it would be interesting to explore in depth the toxins that are present within the venom and to study their potential as calcium/sodium/potassium channels blockers especially since many channelopathies arise from mutations in these channels. In addition, the investigation of the general consequences of *M. bornmuellei’s* toxins on the nervous system can also be envisaged.

## 5. *Macrovipera lebetina*

*Macrovipera lebetina* (Figure 1B) is one of the four currently recognized species of the *Macrovipera* genus. *Macrovipera* genus are terrestrial, oviparous, and venomous vipers that are not only geographically located in the Middle East, but also in North Africa, Near East as well as Milos island in the Aegean sea, where the vipers live in semi-deserts and steppes [64] (Figure 1D). Compared to *Montivipera bornmuelleri*, the snake, *Macrovipera lebetina*, is relatively large, as the females can reach a total length of 150 cm [65].

### 5.1. Venom Composition

Detailed characterization of venoms of some *Macrovipera lebetina* subspecies, such as *Macrovipera lebetina obtusa* and *Macrovipera lebetina transmediterranea*, are available. The venom proteins belong to two major families of enzymes and non-enzymatic proteins. Identified enzymes include serine proteinases, metalloproteinases, l-AAO, phospholipase A2, and hyaluronidase [64,66,67]. Proteins without enzymatic activity consist of disintegrins, C-type lectin proteins (CLPs), natriuretic peptides, myotoxins, CRISP toxins, nerve and vascular endothelial growth factors (NGF/VEGF), cystatin, and kunitz-type proteinase inhibitors [64,67]. A comparison of the venom composition between *Macrovipera lebetina obtusa/euphratica* (Turkey) and *Macrovipera lebetina lebetina* (Cyprus island) showed a more complex venom composition in *Macrovipera lebetina obtusa/euphratica*, likely due to their larger territory and adaptation to different habitats [67].

### 5.2. Venom Biological Activities

The characterization of *Macrovipera lebetina* venom included a detailed description of venom content and biological activities. For example, metalloproteinase from *Macrovipera lebetina transmediterranea* venom was identified as a myotoxin causing muscle degeneration by affecting myofiber stability and disrupting interactions of myofibers with extracellular matrix components, such as laminin and fibronectin [68]. *Macrovipera lebetina* venom was also described as an extraordinarily valuable source of antibacterial, antifungal, and anti-neoplastic compounds [69]. Interestingly, the venom from different *Macrovipera lebetina* subspecies differ in their antiproliferative and antimicrobial efficiency against different cancer cell lines or bacterial and fungal species. This observation is likely due to variable compositions of venoms, including metalloproteinases, l-AAO, and PLA2 in the different subspecies [67]. *Macrovipera lebetina* venom was also able to inhibit adhesion of distinct melanoma and colon adenocarcinoma cells to the extracellular matrix. In fact, PLA2, C-type lectin-like, and kunitz inhibitors prevent cell adhesion via integrins and receptors of the extracellular matrix (ECM) that play a vital role in pathophysiological processes by binding to ECM ligands, such as collagens, to mediate wound healing, tumor metastasis, and thrombosis [70]. *Macrovipera lebetina* PLA2 (MVL-PLA2) shows anti-integrin (α5β1 and αvβ3) properties, inhibiting tumor cell adhesion and migration in vitro as well as angiogenesis in vitro and in vivo. The negative impact of MVL-PLA2 on angiogenesis is mainly due to its potential against integrin αvβ3, known to be involved in angiogenesis. One of the most important strategies in cancer therapy is targeting adhesion and migration of human microvascular-endothelial cells (HMEC-1) that are inhibited by phospholipases, likely through an increase in microtubule dynamics and reorganization of the actin cytoskeleton [71,72]. Together, these findings suggest MCL-PLA2 as a potential anticancer bioactive molecule that requires further investigation. Lebein, a disintegrin from *Macrovipera lebetina’s* venom, is found to have anti-platelet activity and fights melanoma and colon cancer by acting on diverse biological pathways [73,74]. In fact, Lebein induces apoptosis in melanoma cells by reactive oxygen species (ROS) generation and activation of the caspase-independent apoptotic pathway. Moreover, lebein reduces proliferation and increases the differentiation of melanoma cells by upregulating microphthalmia-associated transcription factor (MITF) through inhibition of extracellular signal-regulated kinase (ERK) phosphorylation. Lebein also upregulates E-cadherin expression, consistent with a reduction in invasive properties [74], and decreases colon cancer cell viability by inducing both caspase-dependent and caspase-independent apoptotic pathways. The disintegrin also limits cell migration through the downregulation of α5β1 integrin and limits neovascularization by reducing vascular endothelial growth factor (VEGF) and a VEGF co-receptor, Neuropilin 1 (NRP1), expression [73]. Besides lebein, many compounds with anticancer potential have been described in *Macrovipera lebetina* venoms. For example, Obtustatin, a selective inhibitor of α1β1 integrin, isolated from the venom of *Macrovipera lebetina obtuse*, fights melanoma by restricting vascularization [75] and decreases the size of malignant sarcoma in mice [76]. Leberagin-C, a disintegrin-like/cysteine-rich protein isolated from *Macrovipera lebetina transmediterranea* venom, inhibits cell adhesion mainly through αvβ3 integrin, and also inhibits αvβ1 and αvβ6 integrins, but to a lesser extent. Based on the integrins the protein interacts with, leberagin-C is considered as an anti-angiogenic molecule. Besides its anti-melanoma cell adhesion properties, Leberagin-C shows an anti-platelet aggregation potential [77].

C-type lectin proteins, other compounds found in the venom of *Macrovipera lebetina*, show interesting and diverse therapeutic potentials. The three C-type lectins, lebecin, lebectin, and lebecetin, display anti-neoplastic as well as anti-platelet properties. Lebectin decreases platelet aggregation via its selective binding to the platelet, glycoprotein Ib (GPIb). The three lectins prevent tumor cell proliferation, adhesion, migration, and invasion by inhibiting integrin family adhesion receptors. Unlike previously characterized venom C-type lectins that inhibit collagen receptor, α2β1 integrin, lebectin and lebecetin inhibit α5β1 and αv-containing integrins [78,79,80,81,82]. Lebectin also shows anti-angiogenic activity in vitro and in vivo [83], and selectively inhibits actively proliferating vascular endothelial cells in choroidal neovascularization of cultured aortic and choroidal explants from mice [84]. Moreover, Lebectin modulates cell-cell contacts mediated by N-cadherin through the PI3K/AKT pathway [85]. Macrovipecetin, another C-type lectin protein from *Macrovipera lebetina* venom, also displays anti-neoplastic properties and affects αvβ3 integrin [86].

Lebetin 2 (L2) has been recently found in *Macrovipera lebetina* venom. This peptide shows structural similarity to B-type natriuretic peptide (BNP), a cardioprotective hormone, and, therefore, displays cardioprotective properties by stimulating natriuretic peptide receptors in ischemia-reperfusion injuries in isolated Langendorff-perfused rat hearts [87].

Among *Macrovipera lebetina* venom compounds, serine proteinase inhibitors have also been purified and screened for their biological activities. For example, serine proteinase inhibitors isolated from the venom of *Macrovipera lebetina transmedditerranea* display potent anti-neoplastic properties by acting through integrins to decrease human glioblastoma cell adhesion and migration [88]. They also show anti-angiogenic properties by inhibiting vascular endothelial cell adhesion to fibrinogen and fibronectin as well as limiting their migration by affecting microtubule dynamics [89].

Several VEGFs isolated from the venom of *Macrovipera lebetina* have been identified as preferentially binding the VEGF receptor [90,91]. Isolated VEGFs have also been shown to bind to VEGF co-receptors, NRP1 and NRP2. Interestingly, the binding affinity and selectivity differs between the different VEGFs for distinct NRPs, rendering the molecules interesting therapeutic candidates [91].

## 6. *Vipera palaestinae*

*Vipera palaestinae* (Figure 1C), also known as *Daboia palaestinae (D. palaestinae)*, is a viper species endemic to some areas of the Middle Eastern region. Snakes are mainly found in Lebanon, Syria, Jordan, and Palestine (Figure 1D). The snake is characterized by an average total length of 70–90 cm, with the maximum length at 130 cm [65].

### 6.1. Venom Composition

*Vipera palaestinae* venom have shown neurotoxic, cytotoxic, and hemorrhagic properties mainly due to the presence of cytolysins, hemorrhagic components [92], thrombins, and integrin inhibitors [93,94]. Other studies have also pointed to the presence of l-AAO [95] and proteolytic factor [96] in the venom of *Vipera palaestinae*.

### 6.2. Venom Biological Activities

Compared to *Montivipera bornmuelleri* and *Macrovipera lebetina,* the venom of *Vipera palaestinae* is considerably less explored. The crude venom shows mainly two major pharmacological activities, hemorrhagic and neurotoxic. First studies on this venom showed that neurotoxicity results from a synergic activity of different venom components that is lost after purification/fractionation manipulation [92]. Consistent with synergy concept, a lethal toxin was also purified from *Vipera palaestinae* venom and found to be composed of two compounds: Phospholipase A2 and a basic protein. Each constituent alone does not exhibit any toxicity; however, both components are mandatory for lethality of the toxin [97,98]. In vitro studies showed that *Vipera palaestinae* phospholipases A are unable to reach and degrade glycerophospholipids located on erythrocytes to induce their hydrolysis [99]. These findings are similar to those of *M. bornmuelleri* and are consistent with the fact that vipers’ venoms are either hemolytic or neurotoxic. Therapeutic potential of some of the identified molecules in *Vipera palaestinae* venom has also been investigated. As such, integrin antagonists isolated from the crude venom of *Vipera palaestinae* displayed anti-neoplastic properties against cultured melanoma cell lines [93] and effectively inhibited adhesion of several cells to type I collagen as well as cell migration [94].

## 7. Concluding Remarks

Nature has always been a rich source of biological compounds either as plant extracts or algae and animal venoms, some of which have constituted the basis of traditional medicine for decades. However, after the expansion of pharmaceutical companies and the spread of commercial drugs, traditional “homemade” therapies shrunk and became limited to the countryside. Recently, wide-spread reports of drug side effects as well as treatment resistant bacterial-, viral-, and chemo- pathologies have pointed towards the importance of natural compounds in the development of new therapeutic strategies. Among animal venoms, those of scorpions, bees, and snakes are the most studied. Snake venoms are known to contain a wide spectrum of peptides and toxins that help the animal to paralyze and digest their prey. For decades, these venoms have been the subject of thousands of studies revealing their wealth of bioactive molecules with therapeutical benefits. Three main vipers are located in the Middle Eastern region: *Montivipera bornmuelleri*, *Macrovipera lebetina*, and *Vipera (Daboia) palaestinae*. Many studies have investigated the composition of these venoms, revealing unexpected compounds with great potential for the development of drugs against tumor progression, angiogenesis, and cardiovascular diseases (Table 1). Certainly, what is currently known about these viper venom compositions is for sure less than what is really present; as such, many unrevealed peptides and/or toxins are to be discovered. Surprisingly, the effect of *Montivipera bornmuelleri* and *Macrovipera lebetina* venoms on the nervous system have not been studied before and should, therefore, be considered. In fact, vipers’ venoms are known to contain neurotoxins that act by blocking ion channels or receptors. The purification and the identification of these toxins, their structures as well as their mechanisms of action will not only help in improving the antiserum development field, but also will be beneficial for the development of new treatments for many pathologies in which ion channels and/or receptors are disturbed.

## Figures and Tables

**Table 1:** Biological activities of Middle Eastern vipers’ venoms and their bioactive molecules.

Snake	Venom/Molecule	Biological Activities	References
***Montivipera bornmuelleri***	**Crude Venom**	Pro- and anti-coagulant activities Indirect hemolysis of human RBCs Reduction of blood pressure Selective cytotoxicity on benign and malignant cells, but not on non-tumorigenic cells Up-regulates pro-inflammatory cytokines and downregulates anti-inflammatory cytokines Antibacterial and anti-fungal activities	[57] [57] [60] [61] [63] [55]
	PLA2	Antibacterial, hemolytic, anticoagulant, and pro-inflammatory activities	[59]
	l-AAO	Antibacterial activity	[56]
***Macrovipera lebetina***	**Crude venom**	Cytotoxicity against normal and cancer cell lines Antibacterial activity and antifungal activities Inhibits adhesion of melanoma and colon adenocarcinoma cells to ECM Anti-tumor activity	[69] [69] [70] [76]
	Metalloproteinase	Myotoxicity	[68]
	PLA2	Inhibits tumor cell adhesion and migration in vitro Inhibits angiogenesis in vitro and in vivo	[71] [72]
	Lebein	Reduces proliferation and induces apoptosis of melanoma cells Inhibits human colon cancer cells proliferation, migration and angiogenesis	[74] [73]
	Obtustatin	Fights melanoma by restricting vascularization Decreases malignant sarcoma size in mice	[75] [76]
	Leberagin-C	Inhibits cell adhesion and shows anti-platelet aggregation potential	[77]
	Lebecetin	Decreases platelet aggregation and inhibits adhesion of cancer cells	[80,81]
	Lebectin	Anti-angiogenic activities in vitro and in vivo Inhibits adhesion, migration and invasion of human tumour cells	[83] [79]
	Lebecin	Anti-tumor activity against breast cancer cells	[82]
	Macrovipecetin	Anti-neoplastic properties	[86]
	Lebetin 2	Displays cardioprotective properties	[87]
	Serine proteinase inhibitors	Anti-neoplastic and anti-angiogenic properties	[88,89]
***Vipera palaestinae***	**Crude venom**	Hemorrhagic activity and neurotoxicity	[92]
	Integrin antagonists	Anti-neoplastic properties Inhibits cell migration and cell adhesion to type I collagen	[93] [94]
